# Lipocalin (LCN) 2 Mediates Pro-Atherosclerotic Processes and Is Elevated in Patients with Coronary Artery Disease

**DOI:** 10.1371/journal.pone.0137924

**Published:** 2015-09-14

**Authors:** Raghav Oberoi, Eskindir P. Bogalle, Lukas A. Matthes, Harald Schuett, Ann-Kathrin Koch, Karsten Grote, Bernhard Schieffer, Jutta Schuett, Maren Luchtefeld

**Affiliations:** 1 Department of Cardiology and Angiology, Philipps-University Marburg, Marburg, Germany; 2 Department of Cardiology and Angiology, Hannover Medical School, Hannover, Germany; Institute of Biochemistry and Biotechnology, TAIWAN

## Abstract

**Background:**

Lipocalin (LCN) 2 is associated with multiple acute and chronic inflammatory diseases but the underlying molecular and cellular mechanisms remain unclear. Here, we investigated whether LCN2 is released from macrophages and contributes to pro-atherosclerotic processes and whether LCN2 plasma levels are associated with the severity of coronary artery disease progression in humans.

**Methods and Results:**

In an autocrine-paracrine loop, tumor necrosis factor (TNF)-α promoted the release of LCN2 from murine bone-marrow derived macrophages (BMDM) and vice versa. Moreover, LCN2 stimulation of BMDM led to up-regulation of M1 macrophage markers. In addition, enhanced migration of monocytic J774A.1 cells towards LCN2 was observed. Furthermore, LCN2 increased the expression of the scavenger receptors Lectin-like oxidized low-density lipoprotein receptor-1 (LOX-1) as well as scavenger receptor class A-1 (SRA-1) and induced the conversion of macrophages to foam cells. In atherosclerotic lesions of low density lipoprotein receptor-deficient (*ldlr* ^*−/−*^) mice fed a high fat, high cholesterol diet, LCN2 was found to be co-localized with macrophages in the shoulder region of the atherosclerotic plaque. In addition, LCN2 plasma levels were significantly increased in plasma samples of these mice. Finally, LCN2 plasma levels correlated with the severity of coronary artery disease (CAD) in patients as determined by coronary angiography.

**Conclusions:**

Here we demonstrated that LCN2 plays a pivotal role in processes involved in atherogenesis by promoting polarization and migration of monocytic cells and development of macrophages towards foam cells. Moreover, LCN2 may be used as a prognostic marker to determine the status of CAD progression.

## Introduction

Vascular inflammation is long established as the major cause of atherosclerosis and in the form of coronary artery disease (CAD) has its most dangerous manifestation [1]. The inflammatory process is driven by many different cytokines and chemokines and by other pro-inflammatory factors predominantly derived from activated macrophages, but also from endothelial cells and smooth muscle cells within the diseased vessel wall as well as systemically from the liver as acute phase reactants.

In this regard, we recently found Lipocalin (LCN)2 as a potential biomarker for the early inflammatory response secreted from hepatocytes in an interleukin (IL)-6/glycoprotein (gp) 130 dependent manner [1]. In the present study we investigated the impact of LCN2 on macrophages activation, polarization, migration and foam cell formation which are known to be the earliest processes in atherosclerotic plaque development.

LCN2, also known as neutrophil gelatinase-associated lipocalin (NGAL), was originally discovered in leukocytes such as neutrophils and then extensively studied in the kidney [2,3]. It is an adipokine with diverse functional properties ranging from protective functions in chronic inflammation of the airway system paired with bacteriostatic effects within the innate immune response through to a role as a biomarker for renal injury [4,5]. It has been already reported that many pro- and anti-inflammatory cytokines and factors such as lipopolysaccharide (LPS), tumor necrosis factor-α (TNF)-α, IL-1β, IL-6 or IL-17 induce LCN2 expression in a variety of cell types including neutrophils [6], adipocytes [7], epithelial cells [8], hepatocytes [1] and mesangial cells [9].

Additionally, elevated LCN2 levels were observed in different inflamed tissues like white adipose tissue, liver and heart [7,10]. In regard to atherosclerosis, recent evidence suggests that LCN2 plays a crucial role in vascular remodeling and plaque instability [11–13]. However, the expression of LCN2 in macrophages as a key player in atherosclerotic plaque formation has not yet been evaluated. Additionally, even though an association of LCN2 with CAD and coronary events has been postulated in the past [14,15] the underlying molecular mechanism has not been investigated so far.

Therefore we evaluated the impact of pro-inflammatory cytokines and factors like TNF-α, IL-6 and oxidized low density lipoprotein (oxLDL), which have been identified as crucial players in atherosclerotic disease initiation and progression [16–18], on LCN2 release from murine bone marrow derived macrophages (BMDM). In addition, we investigated pro-atherosclerotic effects of LCN2 on murine macrophages such as migratory capacity, macrophage polarization and foam cell formation mimicking the local situation within the early atherosclerotic lesion. Our findings were further correlated with LCN2 plasma levels in atherosclerosis-prone mice as well as in patients with different degrees of CAD severity.

## Material and Methods

### Recombinant proteins and antibodies

Recombinant murine IL-6 was purchased from CellSystems (Troisdorf, Germany), TNF-α from Miltenyi Biotec (Bergisch Gladbach, Germany), LCN2 from Enzo Life Sciences (Lörrach, Germany), macrophage colony-stimulating factor (M-CSF) and platelet-derived growth factor (PDGF) from R&D-Systems (Minneapolis, MN), and human oxLDL from Source Bioscience (Nottingham, UK). F4/80-PE antibody was obtained from eBioscience (San Diego, CA), appropriate immune globulin (Ig)G-PE as well as the biotinylated CD31c antibody was obtained from BD Bioscience (San Jose, CA). LCN2 antibody was purchased from Abcam (Cambridge, UK), MOMA2 antibody was obtained from Acris (Herford, Germany), and iNOS antibody was purchased from Life Technologies (Darmstadt, Germany). Glyceraldehyde-3-phosphate dehydrogenase (GAPDH) antibody was from Santa Cruz Biotechnology (Santa Cruz, CA), arginase (Arg)1 and chitinase 3-like 3 (Ym1) antibody were from R&D Systems. Biotinylated secondary antibody and ImmPRESS HRP Polymer Detection Kit (anti-rat IgG and anti-rabbit) were obtained from Vector Laboratories (Burlingame, USA). Appropriate alkaline phosphatase (AP)-conjugated secondary antibody was purchased from Sigma Aldrich (Seelze, Germany). Appropriate horseradish peroxidase (HRP)-conjugated secondary antibodies were from GE Healthcare (Munich, Germany).

### Study population

98 patients undergoing coronary angiography at the Department of Cardiology at Hannover Medical School were analyzed. The study was performed in accordance with the Helsinki Declaration and approved by the ethical Committee of Hannover Medical School (#224–2007) and patients provided written informed consent. Patient characteristics are summarized in S1 Table.

### Mice and cells

Male C57BL/6J and C57BL/6J *ldlr* ^*−/−*^ (B6.129S7-Ldlr^*tm1Her*^/J) were obtained from the Jackson Laboratory (Bar Harbor, ME) and maintained under pathogen-free conditions in the Central Animal Facility at Hannover Medical School. All experiments were approved by the Institutional Animal Care (Central Laboratory Animal Facility and Institute for Laboratory Animal Science) and the governmental animal ethics committee (Lower Saxony State Office for Consumer Protection and Food Safety, Germany, permit numbers 06/1189 and 4/39), and performed according to the guidelines of the Federation of European Animal Science Associations. All efforts were made to minimize suffering. At 10 to 12 weeks of age, male C57BL/6J *ldlr* ^*−/−*^ mice were randomly assigned to two groups, one group was subjected to a high fat, high cholesterol diet (D12108, Research Diets, New Brunswick, NJ) for 24 weeks, the other group was subjected to normal chow diet. Subsequently, mice were euthanized by cervical dislocation under deep isoflurane (2%) anesthesia and tissue and plasma was collected.

To generate murine BMDM, bone marrow cells from the femurs and tibias of male C57BL/6J were isolated and cultured in RPMI 1640 medium (Invitrogen, Darmstadt, Germany), supplemented with 10% fetal calf serum (FCS, PAA Laboratories, Cölbe, Germany), 1% penicillin/streptomycin (P/S, PAA Laboratories) and M-CSF (25 ng/mL) for 7 days. Mature macrophages were subsequently tested for the expression of the macrophage marker F4/80 by flow cytometry (S1 Fig). Prior to stimulation, cells were serum-starved for 18 hours in RPMI 1640 medium supplemented with 1% FCS and 1% P/S.

J774A.1 cells were cultured in RPMI 1640 medium with 10% FCS and 1% P/S. For starvation cells were kept for 18 hours in RPMI medium supplemented with 1% FCS and 1% P/S.

Smooth muscle cells (SMC) were isolated from the aorta of male C57BL/6N mice by an enzymatic dispersion method as described before [19]. Cells were cultured on collagen-coated flasks in Dulbecco's modified Eagle's medium (DMEM, Biochrom, Berlin, Germany) containing 1.0 g/L glucose supplemented with 10% FCS (PAA) and 1% P/S (PAA). SMC between passage #2 and passage #5 were used for successive experiments.

### Real-time polymerase chain reaction (PCR)

Total RNA from primary macrophages was isolated using TriFast-Reagent (peqLAB, Erlangen, Germany) and reverse-transcribed with SuperScript reverse transcriptase (Invitrogen), oligo(dT) primers and deoxynucleoside triphosphates. Real-time PCR was performed in duplicates in a total volume of 12.5 μL using brilliant SYBR Green PCR master mixture (Stratagene, Agilent Technologies, Waldbronn, Germany) on a Step One Plus Real-Time PCR system (Applied Biosystems, Darmstadt, Germany) in 96-well PCR plates (Applied Biosystems). Real-time PCR was done with an initial denaturation step at 95°C for 10 min followed by standard 40 PCR cycles consisting of 95°C for 15 s, 60°C for 1 min, 72°C for 1 min and SYBR Green fluorescence emission were monitored after each cycle. For normalization, duplicates of hypoxanthine phosphoribosyltransferase (HPRT) expression were determined in parallel. Relative gene expression was calculated using the 2^–ΔΔCT^ method depicting the n-fold change vs. control [20]. PCR primers were obtained from MWG Biotech (Ebersberg, Germany) and primer sequences are given in S2 Table.

### Flow cytometry

Differentiation of BMDM was analysed using a FACSCalibur flow cytometer (BD Biosciences). Cells were stained with F4/80-PE antibodies (2 μg/mL) and appropriate IgG-PE (2 μg/mL) was used as isotype control (S1 Fig).

### Enzyme-linked immunosorbent assay (ELISA)

Commercial ELISAs from R&D Systems were performed according to the manufacturers’ protocols and analyzed by a μQuant plate reader (Bio-Tek Instruments, Winooski, VT). Supernatants from primary macrophages were analysed for LCN2 after oxLDL, IL-6 or TNF-α stimulation or for TNF-α after LCN2 stimulation. Plasma samples obtained from *ldlr* ^*−/−*^ mice after 24 weeks on high fat, high cholesterol diet or chow diet (control) as well as plasma samples from patients with documented CAD were analysed for LCN2.

### Western blot

Proteins from cellular extracts of BMDM were separated by denaturing SDS-PAGE (10%), and transferred to a PVDF membrane (GE Healthcare). Transferred proteins were probed with antibodies against LCN2 (1:350), Arg1 (1:2000), Ym1 (1:1000) and GAPDH (1:2000). Visualization was accomplished using appropriate peroxidase-conjugated secondary antibodies (1:2000), ECL solution (Bio-Rad Laboratories, Hercules, CA), and an image analysis system (Intas Science Imaging Instruments, Göttingen, Germany). Densitometrical analysis was performed with the help of the software Quantity One (Bio-Rad Laboratories).

### Migration

Evaluation of cell migration of J774A.1 cells was performed using transwell cell culture inserts (8 μm pore size; Corning Life Sciences, Amsterdam, The Netherlands). Cells were cultured in RPMI 1640 medium supplemented with 1% FCS for 24 hours. 100,000 cells were seeded in the upper chamber of the transwell system in RPMI1640 medium. The lower chamber contained RPMI1640 medium supplemented with either 0.5 μg/mL LCN2 or 10% FCS (control). Migration was carried out at 37°C and 5% CO_2_ in a humidity chamber. After 24 hours, pictures of adherent cells on the bottom of the lower compartment were taken with a digital camera connected to an inverted cell culture microscope (CKX41, Olympus, Hamburg, Germany). Cell number per picture was determined by computer-assisted morphometric analysis (ImageJ, NIH, Bethesda, MD, USA) and presented as migration over control.

### Foam cell formation

Foam cell formation was analyzed by the ability of primary macrophages to take up oxLDL labelled with the lipophilic tracer Dil (1,1´-dioctadecyl-3,3,3`,3`-tetramethylindocarbo-cyanine perchlorate, Intracel Resources, Frederick, MD). Therefore, M-CSF generated BMDM were cultured under serum-free conditions for 14 hours in RPMI 1640 medium and either stimulated with LCN2 (0.5 μg/mL) for 6 hours or remained unstimulated followed by an incubation with Dil-labelled oxLDL (10 μg/mL) for 4 hours in RPMI 1640 supplemented with 1% FCS. Foam cell formation was evaluated using an Olympus IX81 fluorescence microscope and data was analyzed by ImageJ software and given as percentage of high power field.

### Proliferation

Proliferation of smooth muscle cells was measured by incorporation of the nucleoside analog 5-bromo-2’-deoxyuridine (BrdU). 10,000 smooth muscle cells were seeded in collagen-coated 96-well plates in DMEM containing 10% FCS and cultured for 24 hours. Subsequently, cells were starved in serum-free medium without any supplements for 2 hours and after changing the medium for additional 24 hours in the presence of 0.5 μg/mL LCN2 or 50 ng/mL PDGF. Proliferation was carried out at 37°C and 5% CO_2_ in a humidity chamber. BrdU was added to the cell culture for the last 4 hours of the stimulation. After cell lyses, the amount of BrdU incorporation was determined with a commercial colorimetric quantification kit (Roche, Mannheim, Germany) according to the manufacturer’s protocol by measuring the absorbance at 450 nm with a plate reader (μQuant; Bio-Tek Instruments).

### Immunohistochemistry and Immunofluorescence

The aortic roots of *ldlr* ^*−/−*^ mice fed a high fat, high cholesterol diet for 24 weeks were dissected and embedded in Tissue Tek OCT^TM^ (Sakura Finetek, Staufen, Germany). Within the aortic root, serial cryostat sections (6 μm, CM3050S, Leica Microsystems, Wetzlar, Germany) at the level of all 3 cusps were prepared. Lesion areas were analyzed for LCN2, endothelial cells (CD31), alpha smooth muscle cells (αSMA), monocytes/macrophages (MOMA-2) as well as the M1 macrophage marker inducible nitric oxide synthase (iNOS) and counterstained with hematoxylin (Carl Roth, Karlsruhe, Germany) for immunohistochemistry or 4',6-diamidino-2-phenylindole (DAPI, Life Technologies) for immunofluorescence, respectively. Visualization was performed with the appropriate VECTASTAIN ABC reagents for biotinylated antibodies and either ImmPACT DAB or VIP Peroxidase (HRP) Substrate (all from Vector Laboratories) according to the manufacturer’s protocol. For immunofluorescence Alexa Fluor 488 and 555 conjugated secondary antibodies were used (Life Technologies). Staining for collagen (Picrosirius Red) were assessed under polarized light. Pictures were captured using a DM4000 B microscope with a DFC320 camera for immunohistochemistry and a DFC350FX camera for immunofluorescence and QWin software (all Leica Microsystems).

### Statistical analysis

All data are presented as the mean±SEM or median with 25^th^/75^th^ percentiles. After testing for normality and equal variance, data were compared using the 2-tailed Student t test for independent samples or one-way ANOVA followed by Fisher´s LSD Post-hoc analysis when more than two groups were examined (SigmaStat 3.0, Systat Software, Erkrath, Germany). A probability value of less than 0.05 was considered statistically significant. Numbers of replicated experiments or animals are indicated in each figure legend. RT-PCR and ELISA measurements were performed in duplicates.

## Results

### TNF-α enhances LCN2 expression and secretion in macrophages

**Fig 1:**
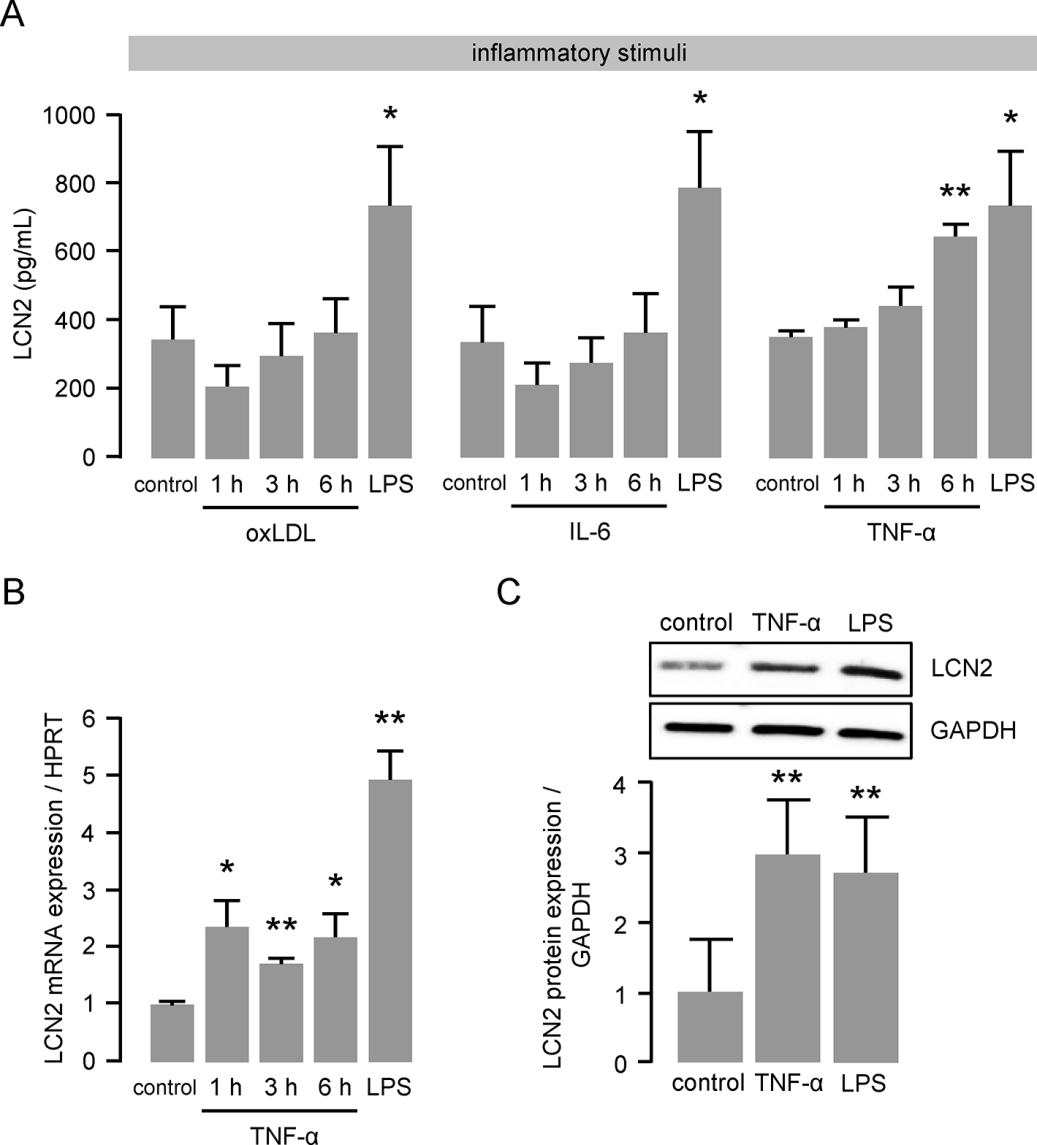
TNF-α enhances LCN2 expression and secretion in macrophages. (**A**) LCN2 release from murine primary BMDM following oxLDL (50 μg/mL), IL-6 (200 ng/mL) or TNF-α (50 ng/mL) stimulation was determined by ELISA at the indicated time points. LPS (100 ng/mL for 6 hours) was used as positive control. *P<0.05, **P<0.01 vs. control, n = 6–8 replicated experiments. (**B**) LCN2 mRNA expression following TNF-α (50 ng/mL) stimulation was determined by real time PCR at the indicated time points. LPS (100 ng/mL for 6 hours) was used as positive control. HPRT was used as housekeeping gene and the relative mRNA expression was calculated using the 2^-ΔΔCT^ method. *P<0.05, **P<0.01 vs. control, n = 4–7 replicated experiments. (**C**) LCN2 protein expression following TNF-α (50 ng/mL) stimulation for 6 hours was determined by Western blot. LPS was used as positive control. GAPDH was used as loading control. **P<0.01 vs. control, n = 5 replicated experiments.

In the early stages of atherosclerosis, TNF-α release from macrophages is a crucial initiation step that leads to the disruption of endothelial junctions and the facilitation of monocyte transmigration [21]. We therefore investigated the impact of TNF-α as an early inflammatory mediator on LCN2 induction in macrophages. In addition, we used IL-6 as a pleiotropic cytokine that is responsible for the induction of hepatocellular acute phase proteins in immune responses and oxLDL as a major contributor to atherosclerotic plaque formation. While stimulation of BMDM with IL-6 and oxLDL did not induce LNC2 release, TNF-α resulted in enhanced LCN2 protein release after 6 hours of treatment (Fig 1A). In addition, we found significantly up-regulated LCN2 mRNA expression (Fig 1B) and protein expression after TNF-α stimulation. LPS was used as positive control for all experiments.

### LCN2 induces migration of monocytic cells

**Fig 2:**
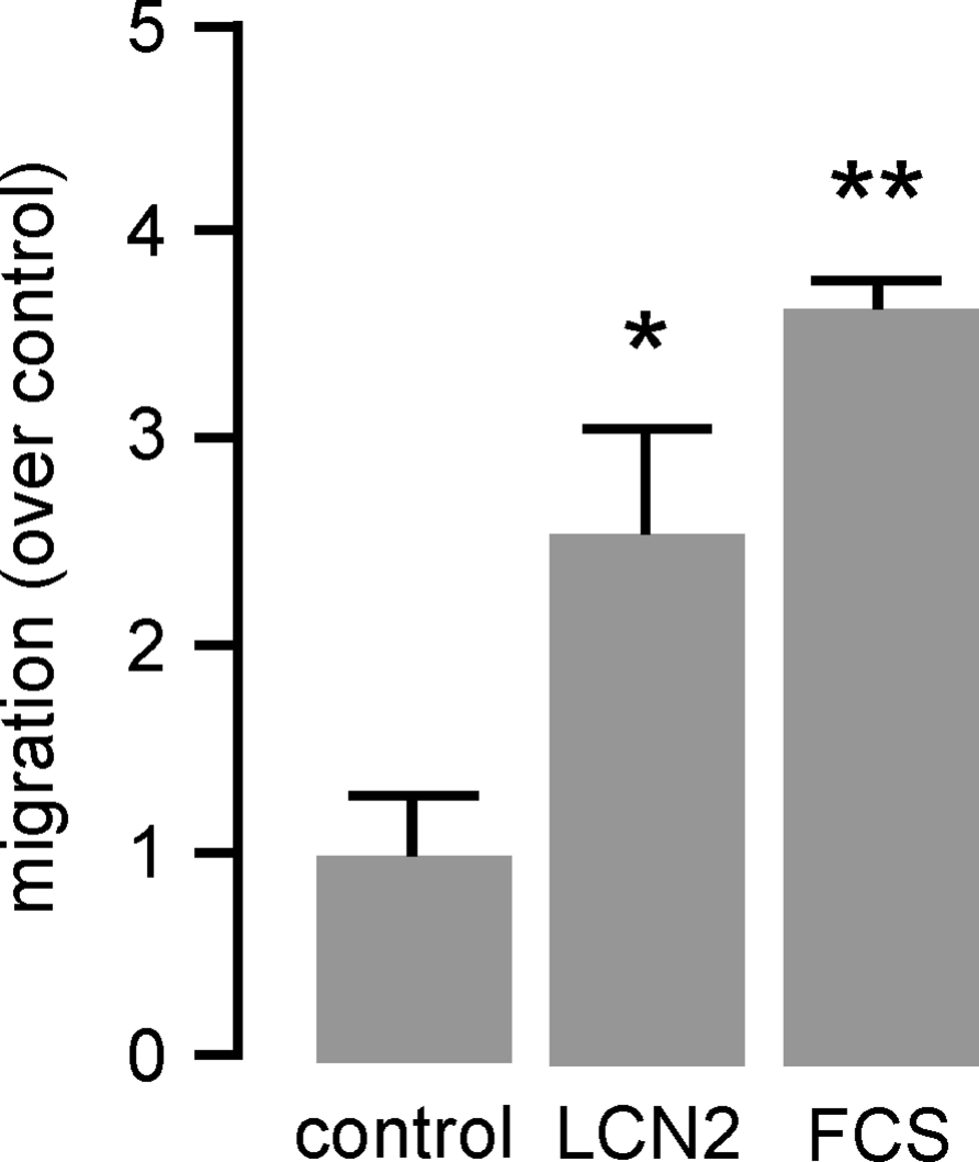
LCN2 induces cell migration of monocytic cells. Migration of murine monocyte/macrophage-like J774A.1 cells in response to LCN2 (0.5 μg/mL) was carried out in transwell cell culture inserts for 24 hours. Medium with 10% FCS was used as positive control. Migration is depicted as induction over unstimulated control. *P<0.05, **P<0.01 vs. control, n = 5 replicated experiments.

Since migration of monocytic cells to the inflamed vessel is decisive for the initial plaque formation, we investigated the capability of LCN2 to impel migratory capacity by using transwell inserts. However, M-CSF-differentiated mature BMDM did not migrate towards LCN2 (data not shown). In contrast, migratory capacity of murine J774A.1 cells was found to be enhanced by LCN2 about ~2.5-fold after 24 hours (Fig 2) pointing to a selective impact of LCN2 to attract monocytes.

### LCN2 enhances the mRNA expression of M1 macrophage markers

**Fig 3:**
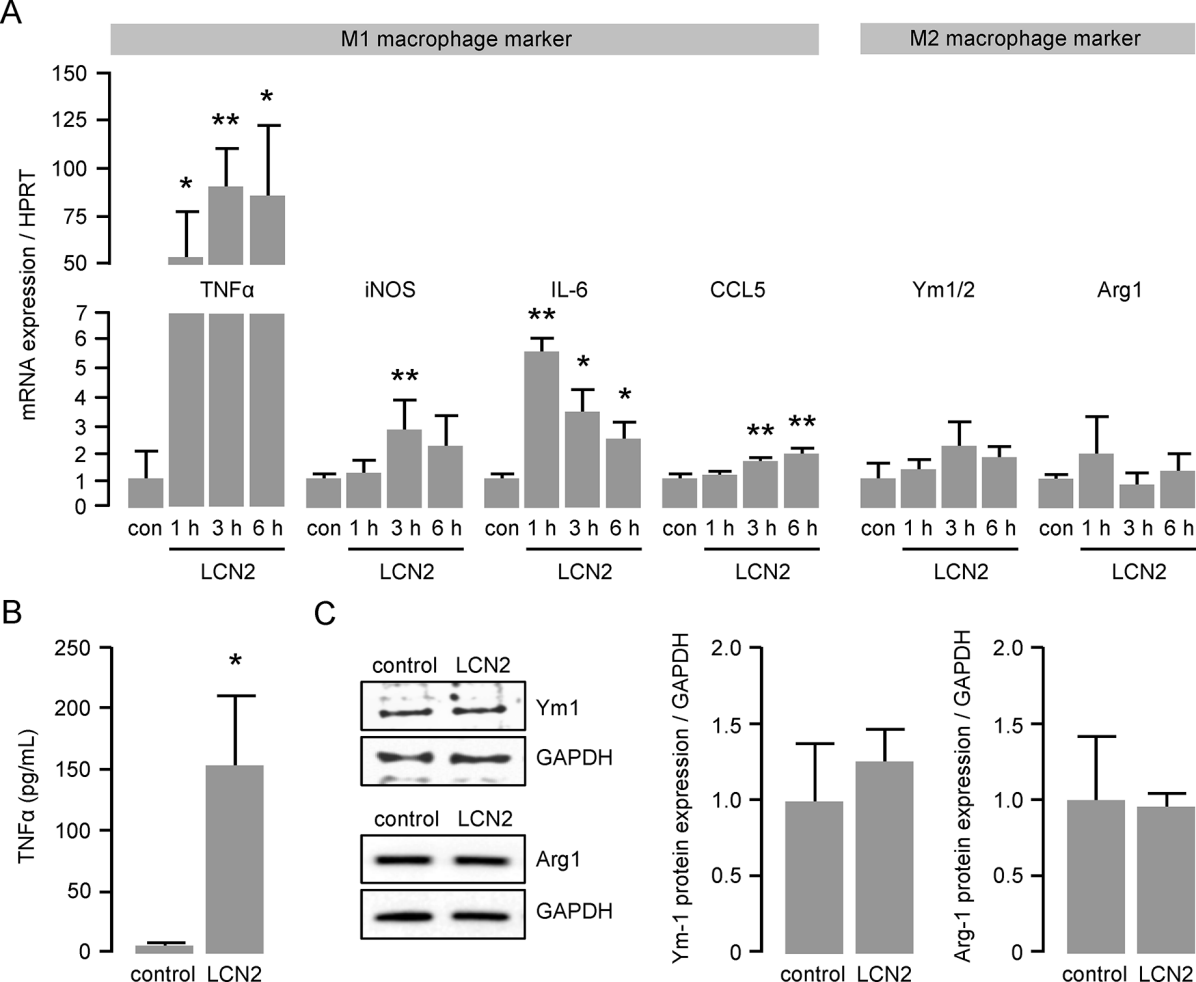
LCN2 enhances mRNA expression of M1 macrophage markers. (**A**) mRNA expression of the M1 macrophage markers TNF-α, iNOS, IL-6 and CCL5 as well as the M2 macrophage markers Ym1/2 and Arg1 in murine primary BMDM following LCN2 (0.5 μg/mL) stimulation was determined by real time PCR at the indicated time points. HPRT was used as housekeeping gene and the relative mRNA expression was calculated using the 2^-ΔΔCT^ method. con = control. *P<0.05, **P<0.01 vs. control, n = 5–10 replicated experiments. (**B**) Release of TNF-α from murine primary BMDM following LCN2 (0.5 μg/mL) stimulation for 6 hours was assessed by ELISA. *P<0.05 vs. control, n = 12. (**C**) Protein expression of the M2 macrophage markers Ym1 and Arg1 following LCN2 (0.5 μg/mL) stimulation for 6 hours was determined by Western blot. GAPDH was used as loading control. n = 3–4 replicated experiments.

Once recruited to the inflammatory site, monocytes transmigrate into the vessel wall where they differentiate into macrophages. To determine the impact of LCN2 on macrophage polarization, we performed real-time PCR analysis for well-established M1 and M2 macrophage markers using RNA isolated from LCN2-treated BMDM. We observed a more than 50-fold induced mRNA expression of the pro-inflammatory M1 macrophage marker TNF-α as early as 1 hour after LCN2 stimulation (Fig 3A). In addition, mRNA expression of other M1 macrophage marker, such as inducible NO synthase (iNOS), IL-6 and Chemokine (C-C motif) ligand (CCL)5 was likewise significantly up-regulated peaking between 1 and 6 hours after LCN2 stimulation (Fig 3A). In contrast, mRNA expression of classical markers of an anti-inflammatory M2 macrophage phenotype such as chitinase 3-like 3 and 4 (Ym1/2) and arginase (Arg)1 were not affected by LCN2 stimulation (Fig 3A). M1 polarization was further confirmed by ELISA showing sustainably enhanced TNF-α levels in the supernatant of BMDM after 6 hours of LCN2 stimulation (Fig 3B). Unchanged expression of the M2 macrophage markers Ym1 and Arg1 after LCN2 stimulation was confirmed by Western blot (Fig 3C).

### LCN2 induces foam cell formation and modulates scavenger receptor expression in macrophages

**Fig 4:**
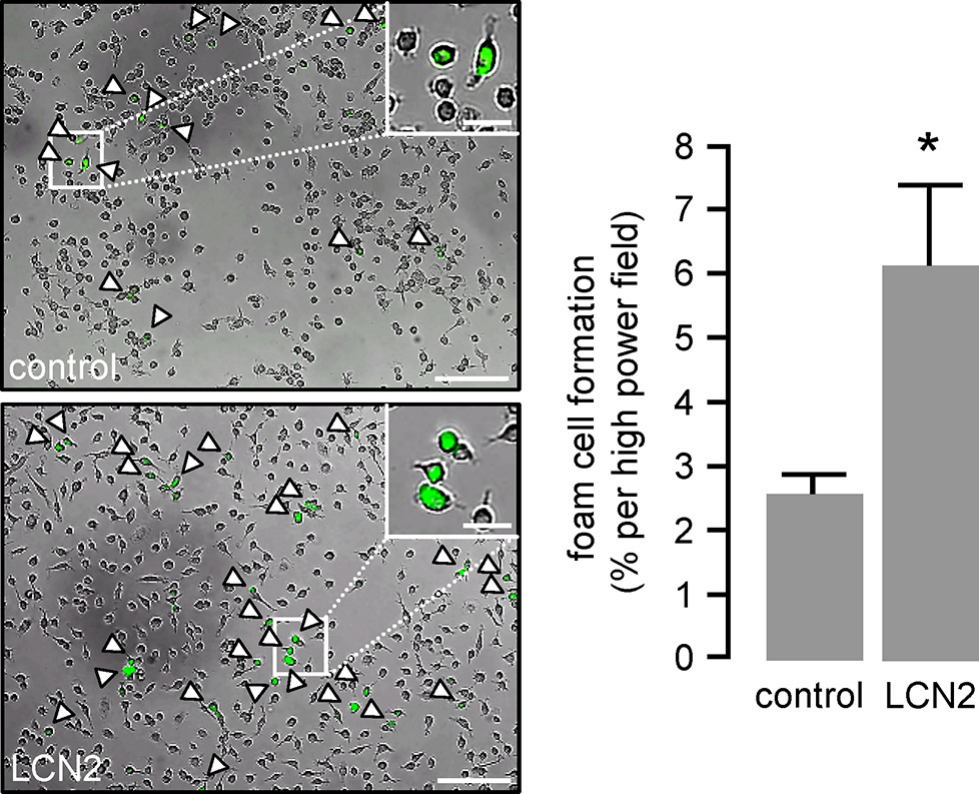
LCN2 induces foam cell formation. Foam cell formation is illustrated by the uptake of Dil-labelled oxLDL (10 μg/mL) for 4 hours. Murine primary BMDM were pre-stimulated with LCN2 (0.5 μg/mL) for 24 hours. Foam cell formation is given as percentage of Dil-positive cells per high power field. Representative pictures are shown. Scale bars = 200 μm. Inserts show a higher magnification of the pictures. Scale bars in insert = 50 μm. *P<0.05 vs. control, n = 6 replicated experiments.

Next we assessed the potential of LCN2 to induce foam cell formation by promoting oxLDL uptake through resident macrophages. Pre-incubation of BMDM with LCN2 over a period of 24 hours significantly enhanced the uptake of Dil-labelled oxLDL. Foam cell numbers were estimated as percentage of Dil-positive cells per high power field (Fig 4).

**Fig 5:**
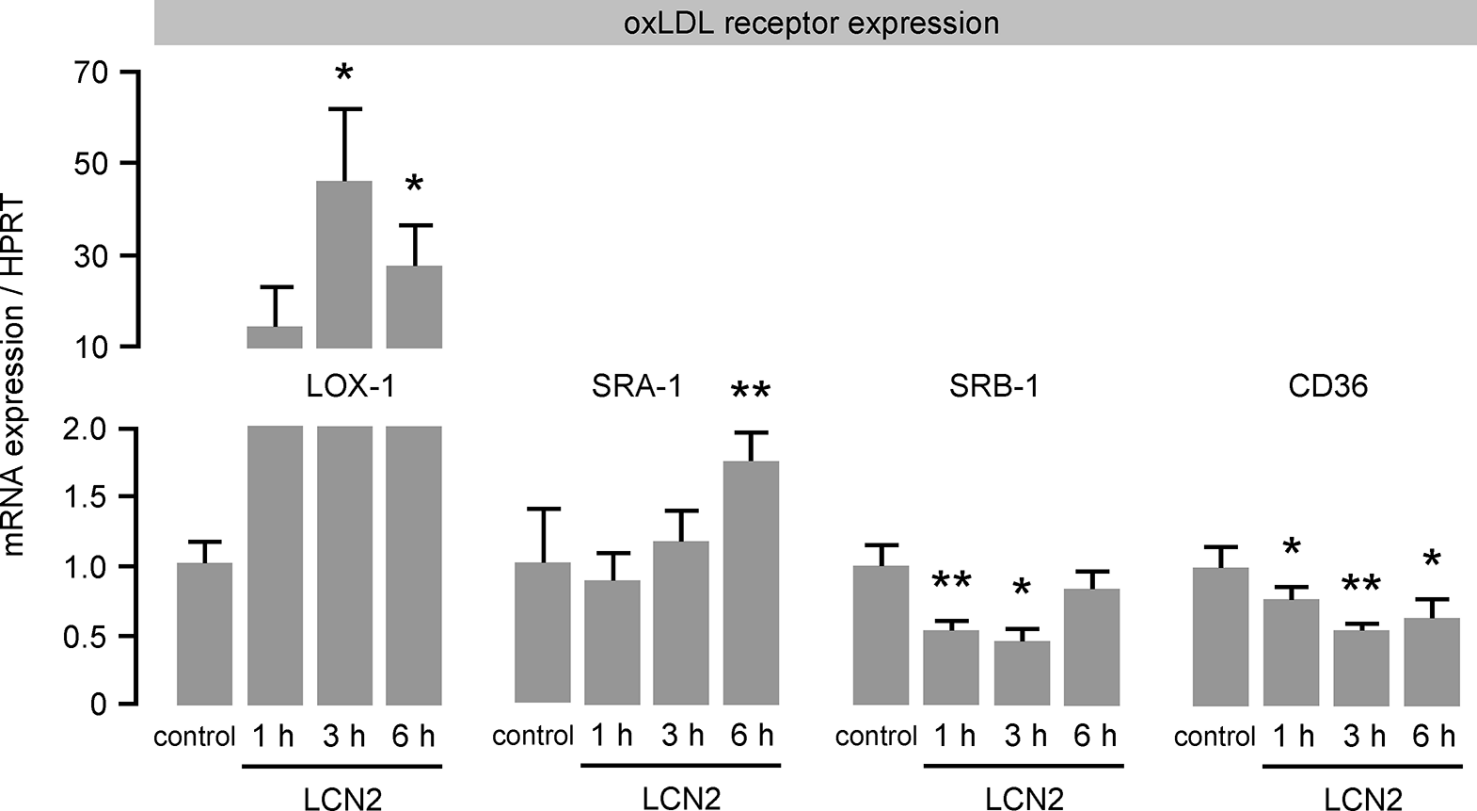
LCN2 modulates the mRNA expression of scavenger receptors in macrophages. The mRNA expression of the oxLDL receptors LOX-1, SRA-1, SRB-1 and CD36 in murine primary BMDM following LCN2 (0.5 μg/mL) stimulation was determined by real time PCR at the indicated time points. HPRT was used as housekeeping gene and the relative mRNA expression was calculated using the 2^-ΔΔCT^ method. *P<0.05, **P<0.01 vs. control, n = 6–10 replicated experiments.

To clarify the mechanism behind augmented oxLDL uptake, we investigated mRNA expression of scavenger receptors in response to LCN2 by real-time PCR. Interestingly, mRNA expression of lectin-like oxLDL (LOX)-1 was found to be highly up-regulated (~50-fold) 3 hours after LCN2 stimulation and was still significantly elevated 6 hours after treatment (Fig 5). In addition, scavenger receptor class A (SRA)-1 mRNA expression was moderately enhanced by LCN2 6 hours after stimulation. In contrast, mRNA expression of scavenger receptor class B (SRB)-1 and CD36 was significantly down-regulated at most of the investigated time points.

### Hypercholesterolemic mice reveal pronounced LCN2 levels in blood plasma and atherosclerotic lesions

**Fig 6:**
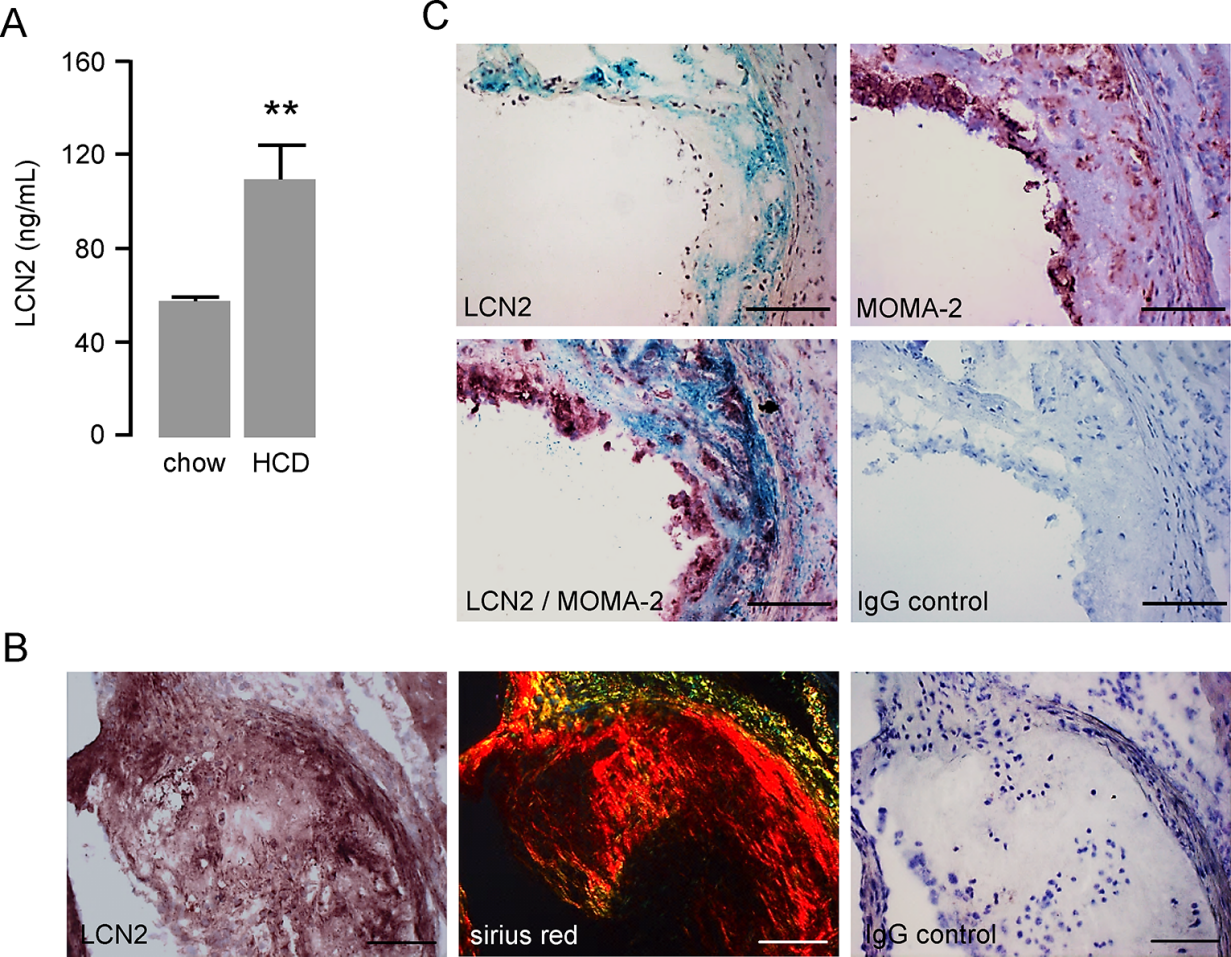
Hypercholesterolemic mice reveal pronounced LCN2 levels in blood plasma and atherosclerotic lesions. (**A**) LCN2 plasma levels of *ldlr* ^*−/−*^ mice fed a high fat, high cholesterol diet (HCD) and of *ldlr* ^*−/−*^ mice fed a normal chow diet (chow) over a period of 24 weeks were quantified by ELISA. **P<0.01 vs. control, n = 5–10 animals. (**B**) Consecutive cross sections of the aortic root dissected from *ldlr* ^*−/−*^ mice 24 weeks after feeding a high fat, high cholesterol diet were stained for LCN2 and Picrosirius Red. For the distinction of different collagen fibres pictures were taken using polarized light. Scale bar = 100 μm. (**C**) Cross sections of the aortic root from *ldlr* ^*−/−*^ mice 24 weeks after feeding a high fat, high cholesterol diet were stained for LCN2 or MOMA-2 or double stained for LCN2 and MOMA-2. Sections were counterstained with hematoxylin. Scale bars = 100 μm. Representative pictures are shown.

To verify our in vitro findings in an atherosclerotic mouse model, we analyzed circulating LCN2 levels in hypercholesterolemic *ldlr* ^*−/−*^ mice fed a high fat, high cholesterol diet for 24 weeks in comparison to control *ldlr* ^*−/−*^ mice fed a chow diet. Of note, LCN2 plasma levels in hypercholesterolemic mice were significantly elevated (55 ng/mL to 110 ng/mL, Fig 6A).

In addition, we determined LCN2 expression patterns in atherosclerotic plaques of the aortic root isolated from *ldlr* ^*−/−*^ mice after high fat, high cholesterol diet by immunostaining. We found LCN2 to be entirely distributed throughout the plaque (Fig 6B and 6C). Interestingly, the strongest LCN2 immunoreactivity was associated with mature collagen type I fibres visualized by Picrosirius Red as orange-red birefringence under polarized light (Fig 6B) indicating accumulated LCN2 in the extracellular matrix particularly in fibrotic regions of the plaque. Further analyses revealed an overlapping staining of LCN2 with cells positive for the general monocyte/macrophage marker MOMA-2 mainly in the shoulder regions of the atherosclerotic lesion (Fig 6C). Analysis for iNOS expression as a prototypical M1 macrophage marker revealed a predominating co-localization with MOMA-2 expression (S2 Fig) demonstrating that the majority of plaque macrophages are M1 macrophages under our experimental conditions of prolonged high fat, high cholesterol feeding.

Additionally we found LCN2 expression within the atherosclerotic lesion to be at least in part co-localized with areas positive for the smooth muscle cell (SMA) marker αSMA (S3A Fig). In contrast, areas stained positive for the endothelial marker CD31 were not overlapping with LCN2 expression (S3B Fig). Since abundant SMA content is another characteristics of early atherosclerotic plaque development, we additionally investigated if LCN2 induce SMA proliferation. However, using BrdU incorporation assays we could not observe any effect of LCN2 on SMC proliferation (S4 Fig).

### Patient with coronary artery disease have higher LCN2 plasma levels

**Fig 7:**
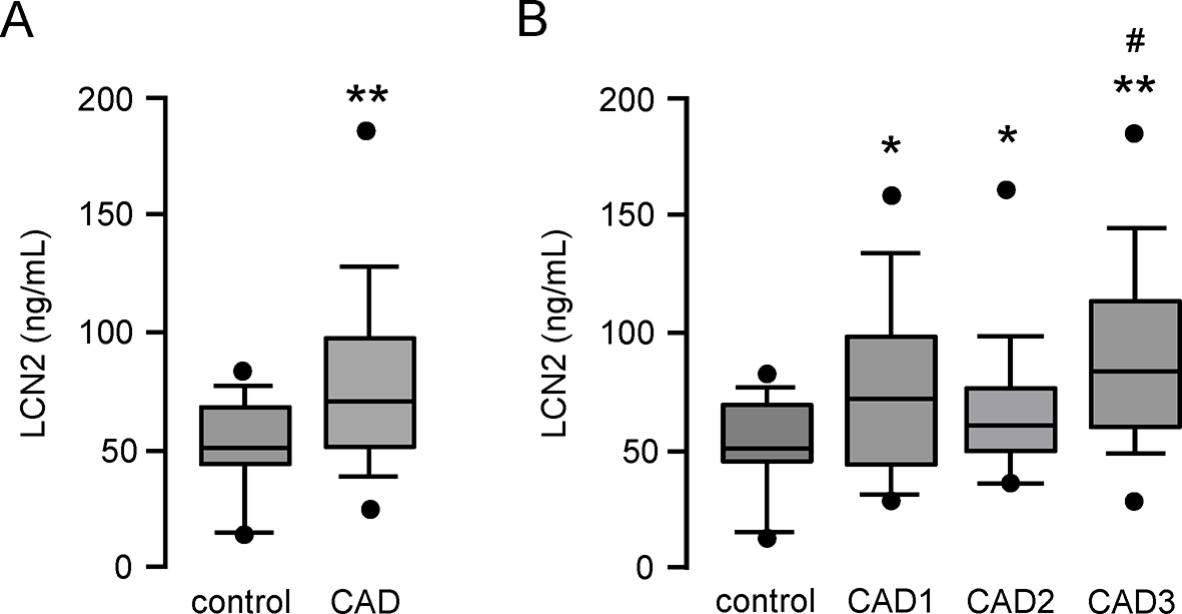
Patients with coronary artery disease have higher LCN2 plasma levels. Plasma samples from 98 patients undergoing coronary angiography were analyzed for LCN2 by ELISA. (**A**) Patients were classified as controls without CAD (n = 20) and with CAD (n = 78). (**B**) Subgroup analysis of control patients without CAD (n = 20) and patients subdivided in 1-vessel (CAD 1, n = 24), 2-vessel (CAD 2, n = 27) and 3-vessel (CAD 3, n = 27) CAD. *P<0.05, **P<0.01 vs. control, ^#^P<0.05 vs. CAD2. Box plots of median with 25^th^/75^th^ percentiles and whiskers with 10^th^/90^th^ percentiles are shown.

In a translational approach, we finally measured LCN2 plasma levels in 98 patients undergoing coronary angiography (for patients characteristics, see S1 Table). In line with recently published data [15,22,23] we found that patients with CAD have significantly higher LCN2 plasma levels as compared to age-matched control patients without CAD (**P<0.01, Fig 7A). Of note, LCN2 plasma levels are approximately gradually increasing with the severity of CAD being highest in patients with severe 3-vessel disease (*P<0.05, **P<0.01, Fig 7B).

## Discussion

Together we here display for the first time that LCN2 is involved in early inflammatory processes which are known to be important for atherogenesis. By inducing monocyte migration, macrophage polarization as well as foam cell formation, LCN2 participates in the main initial events in atherosclerotic plaque development.

In a previous study we reported that hepatic expression of LCN2 is IL-6/gp130-dependent [1]. Here we demonstrate that TNF-α, as an early marker of inflammation but not oxLDL or IL-6, which occurs later in inflammatory processes induces LCN2 expression in macrophages, which was also shown for other cell types e.g. adipocytes [7]. TNF-α as a pro-inflammatory cytokine is primarily produced by monocytes and macrophages. The involvement of TNF-α in atherosclerotic plaque development is supported by several studies [24–26]. Of note, LCN2 stimulation of macrophages also leads to an extensive up-regulation of TNF-α pointing to the formation of a vicious cycle, which may perpetuate the inflammation during atherogenesis. However, since TNF-α in the same way serves as a M1 macrophage marker, we further analyzed the expression of other macrophage polarization markers. In line with enhanced TNF-α expression, we also found the M1 markers iNOS, IL-6 and CCL5 to be significantly increased upon LCN2 stimulation. Since LCN2 is an activator of the NF-κB signaling pathway [10], the induction of NF-κB-dependent genes such as TNF-α and iNOS seems reasonable. Along the same lines, our results are supported by previously published data showing that mice lacking LCN2 showed reduced M1 marker expression in microglia after LPS injection [27]. In addition, Cheng et al. found that LCN2 promotes M1 macrophage polarization, and LCN2 neutralization attenuates cardiac ischemia injury [28]. However, there are conflicting data in regard to macrophage polarization in response to LCN2. Guo et al. reported that lcn2-deficiency up-regulated expression of M1 macrophage marker but down-regulated expression of M2 macrophage marker in adipose tissue and liver of mice upon a high fat diet feeding [29]. Furthermore, Warszawska et al. identified LCN2 as both a marker of deactivated macrophages and a macrophage deactivator which attenuates the early inflammatory response and impairs bacterial clearance [30]. It is therefore tempting to speculate that LCN2 effects are maybe diverse in different inflammatory settings. In our study, we observed scavenger receptor CD36 expression–a marker for M2 macrophages and mostly responsible for macrophage foam cell formation [31,32]–to be down-regulated in this experimental setting. However, we found LCN2 induced foam cell formation. To clarify this contradiction, we analyzed additional scavenger receptors which could be potentially involved in the process of LCN2-induced foam cell formation. Interestingly, we identified LOX-1, which is described as an alternative oxLDL receptor mostly expressed on endothelial cells [33] but also on macrophages [34] and SMA [35], to be extensively up-regulated in macrophages stimulated with LCN2, whereas SRA-1 and SRB-1 receptor expression were only slightly affected. These results point to an alternative mechanism how M1 macrophages could become foam cells, independent of the expression of the classical scavenger receptor CD36. We further investigated whether LCN2 elicits other pro-atherosclerotic features like migration of monocytes into the vessel wall or proliferation of SMC. In the present study we could demonstrate that LCN2 itself acts as a chemoattractant for monocytes. However, stimulation of SMA with LCN2 had no influence on cell proliferation (S4 Fig)–another feature of early atherosclerotic plaque development. Thus we hypothesize that the release of LCN2 from resident macrophages within the vessel wall leads to the migration of monocytes along a chemotactic gradient towards the inflamed tissue where they become polarized M1 macrophages as well as foam cells, which then impel the pro-inflammatory response in the atherosclerotic-prone vessel (S5 Fig).

To support this hypothesis, we further analyzed whether macrophages within the atherosclerotic lesion express LCN2. First of all, we found enhanced LCN2 levels in plasma samples of atherosclerotic prone low density lipoprotein receptor deficient mice fed with a high fat, high cholesterol diet. This finding was correlated with abundant expression of LCN2 within the atherosclerotic lesion, where we found LCN2 to be expressed in macrophage rich areas mainly in the shoulder regions as well as in the media of SMC rich areas as previously described [11]. But mostly we found LCN2 to accumulate in the extracellular matrix in areas of massive collagen deposition. As LCN2 is a secreted protein, it is tempting to speculate that after its release from cells of the atherosclerotic lesion, it accumulates within the collagen-rich extracellular matrix where it exerts its pro-inflammatory effects, like macrophage M1 polarization and foam cell formation. Moreover, recent evidence suggests that LCN2 plays a crucial role in vascular remodelling and plaque instability in atherosclerosis [11,36]. In addition, different studies reveal that LCN2 levels are significantly increased in patients with CAD and correlate with disease severity [37] and myocardial infarction [38] and therefore might serve as a potential biomarker for cardiovascular events. Likewise, we found increasing LCN2 plasma levels in patients with increasing CAD severity. However, we could not ideally document a stepwise increase, likely because of the rather small numbers of patients in our cohorts. Even though a correlation of enhanced LCN2 levels with disease severity could be demonstrated, the underlying mechanism if and how LCN2 is involved in this process remains still unknown. Thus, we speculate, that elevated plasma levels of LCN2 reflecting the enhanced accumulation of LCN2 within the atherosclerotic lesion, leading to macrophage activation, which may be finally responsible for the severity of the CAD. By analysing plasma samples of patients with angiographically documented CAD, we confirmed its potential role as a marker for cardiovascular disease as well as its correlation with the severity of CAD, which may be in part driven by LCN2-activated macrophages.

Together, the present study demonstrates that LCN2 is not only a potent marker for CAD but also a mediator which is involved in different paths during initiation and perpetuation of pro-atherosclerotic processes during plaque development, mainly by macrophage activation. Further analyses to determine whether the observed pro-inflammatory effects of LCN2 are dependent on LCN2-induced TNF-α release should be carried out in future studies.

## Supporting Information

S1 Fig Confirmation of maturation status of primary macrophages. Murine BMDM were analyzed for the macrophage marker F4/80 after differentiation with M-CSF for 7 days. Cell surface expression of F4/80 was verified by flow cytometry using PE-labelled antibodies (filled graph). Appropriate PE-labelled isotype IgG were used as control (open graph). A representative picture is shown.
(PPT)

S2 Fig Consecutive cross sections of the aortic root dissected from *ldlr* ^*−/−*^ mice 24 weeks after feeding a high fat, high cholesterol diet were stained for iNOS or MOMA-2 or double stained for iNOS and MOMA-2. (**A**) Immunohistochemistry and (**B**) immunofluorescence. All stainings were counterstained with hematoxylin or DAPI, respectively. Scale bars = 100 μm. Representative pictures are shown.
(PPT)

S3 Fig Consecutive cross sections of the aortic root dissected from *ldlr* ^*−/−*^ mice 24 weeks after feeding a high fat, high cholesterol diet were stained for (A) LCN2 and α-SMA or for (B) LCN2 and CD31. All stainings were counterstained with hematoxylin. Scale bars = 100 μm. Insert shows a higher magnification of the picture. Scale bar in insert = 25 μm. Representative pictures are shown.
(PPT)

S4 Fig LCN2 does not induce proliferation of SMC. Proliferation of murine SMC in response to LCN2 (0.5 μg/mL) was measured as BrdU incorporation. 50 ng/mL PDGF was used as positive control. Proliferation is depicted as induction over unstimulated control. **P<0.01 vs. control, n = 3 replicated experiments.
(PPT)

S5 Fig Schematic model on pro-atherosclerotic effects of LCN2. (PPT)

S1 Table 98 patients undergoing coronary angiography were classified as controls without CAD or as patients with one- (CAD 1), two- (CAD 2) or three vessel coronary artery disease (CAD 3). Body-mass index is defined as the weight in kilograms divided by the square of the height in meters. Median; 25th/75th percentile, *P<0.05 vs. control, **P<0.01 vs. control, ^§§^P<0.01 vs. CAD1, ^#^P<0.05, ^##^P<0.01 vs. CAD2.
(DOC)

S2 Table Arg1: arginase 1, CCL5: Chemokine (C-C motif) ligand 5, CD36: cluster of differentiation 36, HPRT: hypoxanthine-guanine phosphoribosyltransferase, IL-6: interleukin 6, iNOS: inducible NO synthase, LCN2: lipocalin 2, LOX-1: lectin-like oxidized low density lipoprotein receptor-1, SRA-1: scavenger receptor class A-1, SRB-1: scavenger receptor class B-1, TNF-α: tumor necrosis factor-α, Ym1/2: chitinase 3-like 3 and 4. (DOC)
