# Supplementary figures and images for: Lipocalin (LCN) 2 Mediates Pro-Atherosclerotic Processes and Is Elevated in Patients with Coronary Artery Disease
Source: PLoS One. 2015 Sep 14;10(9):e0137924. doi: 10.1371/journal.pone.0137924 (PMC4569430; doi:10.1371/journal.pone.0137924)

## Slide 1
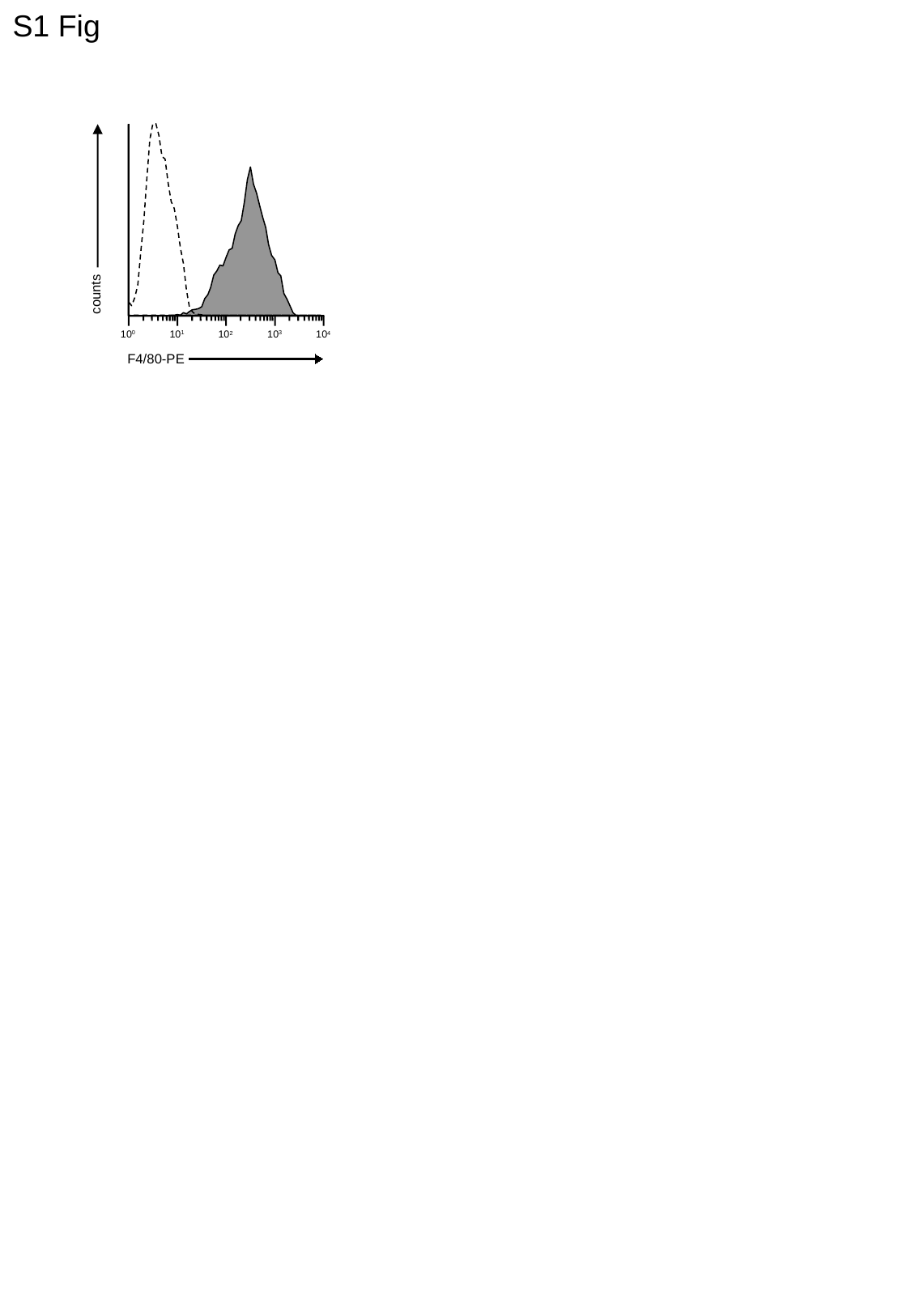

S1 Fig
counts
100
101
103
102
104
F4/80-PE

Supplement: S1 Fig — Murine BMDM were analyzed for the macrophage marker F4/80 after differentiation with M-CSF for 7 days. Cell surface expression of F4/80 was verified by flow cytometry using PE-labelled antibodies (filled graph). Appropriate PE-labelled isotype IgG were used as control (open graph). A representative picture is shown. (PPT) [file pone.0137924.s001.ppt]

## Slide 1
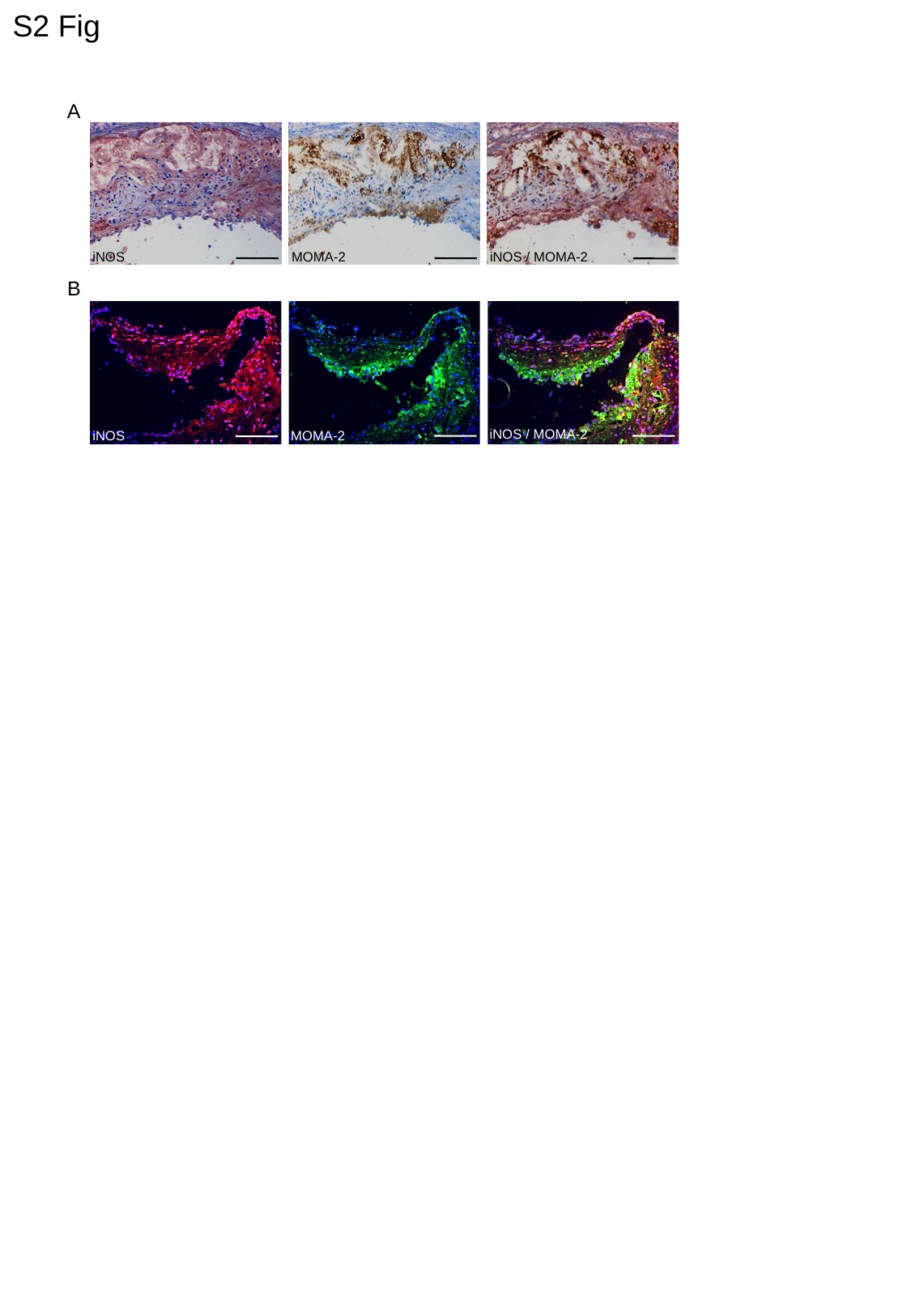

S2 Fig
A
iNOS
MOMA-2
iNOS / MOMA-2
B
iNOS / MOMA-2
iNOS
MOMA-2

Supplement: S2 Fig — (A) Immunohistochemistry and (B) immunofluorescence. All stainings were counterstained with hematoxylin or DAPI, respectively. Scale bars = 100 μm. Representative pictures are shown. (PPT) [file pone.0137924.s002.ppt]

## Slide 1
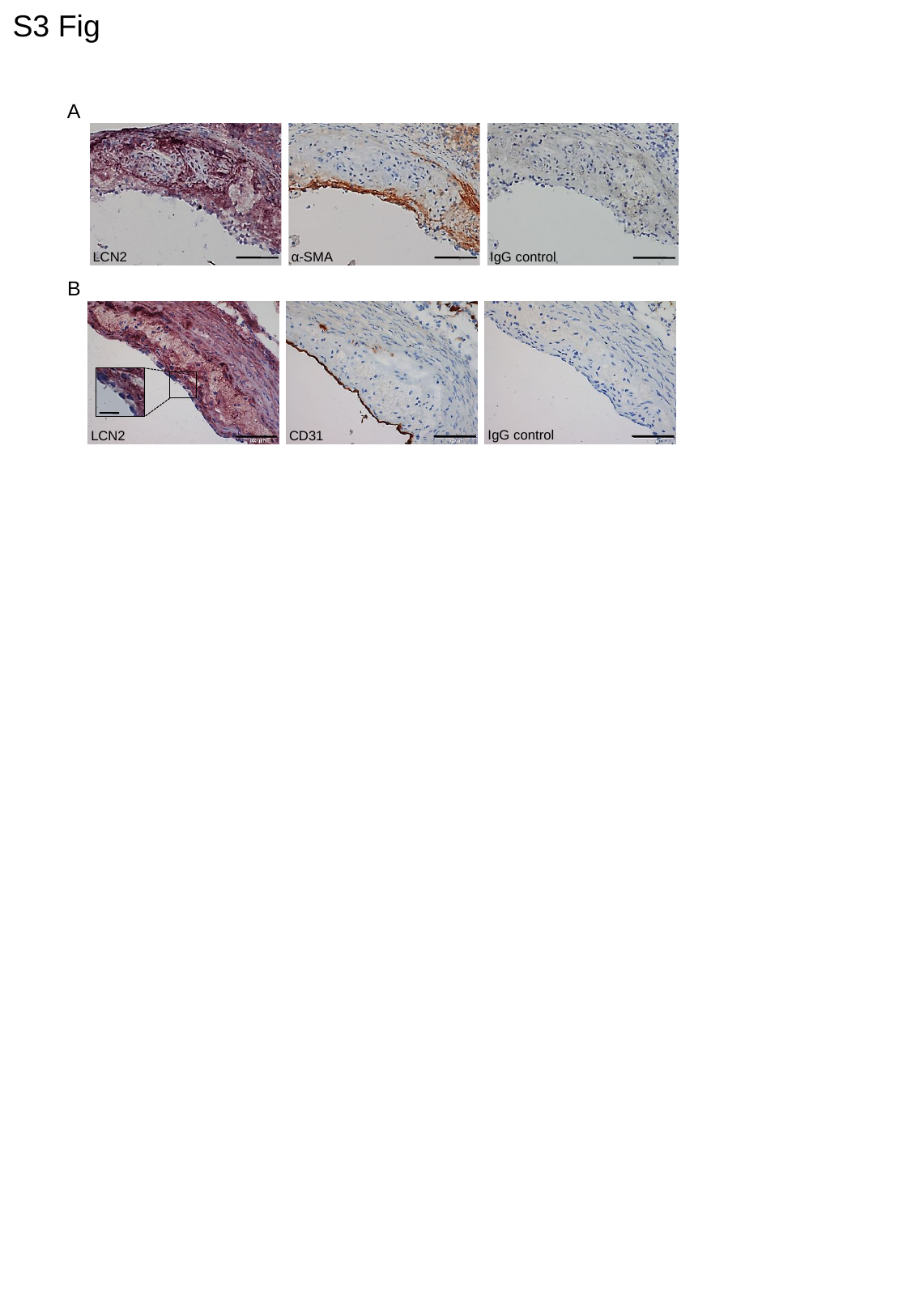

S3 Fig
A
LCN2
α-SMA
IgG control
B
IgG control
LCN2
CD31

Supplement: S3 Fig — All stainings were counterstained with hematoxylin. Scale bars = 100 μm. Insert shows a higher magnification of the picture. Scale bar in insert = 25 μm. Representative pictures are shown. (PPT) [file pone.0137924.s003.ppt]

## Slide 1
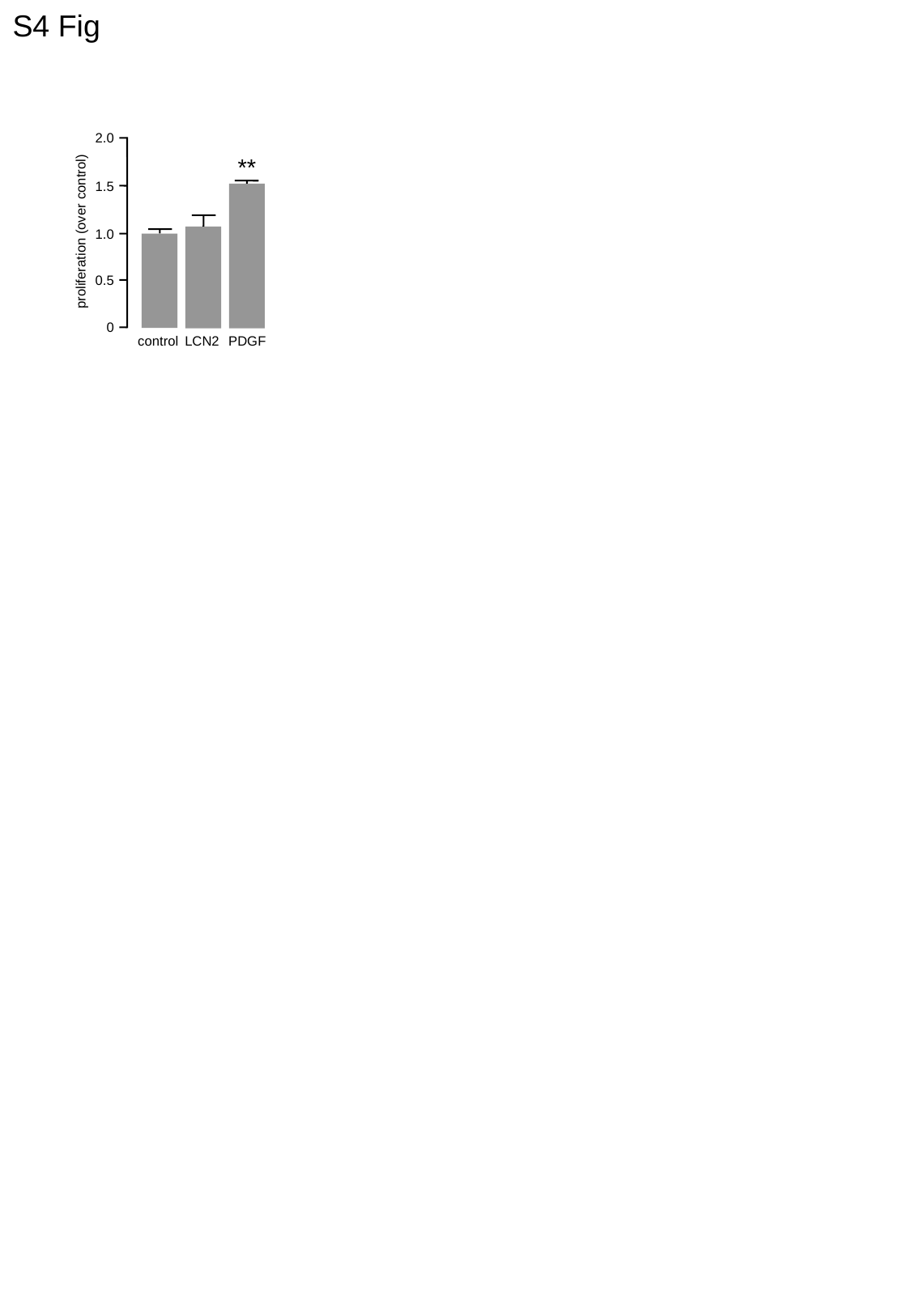

S4 Fig
2.0
**
1.5
proliferation (over control)
1.0
0.5
0
control
LCN2
PDGF

Supplement: S4 Fig — Proliferation of murine SMC in response to LCN2 (0.5 μg/mL) was measured as BrdU incorporation. 50 ng/mL PDGF was used as positive control. Proliferation is depicted as induction over unstimulated control. **P<0.01 vs. control, n = 3 replicated experiments. (PPT) [file pone.0137924.s004.ppt]

## Slide 1
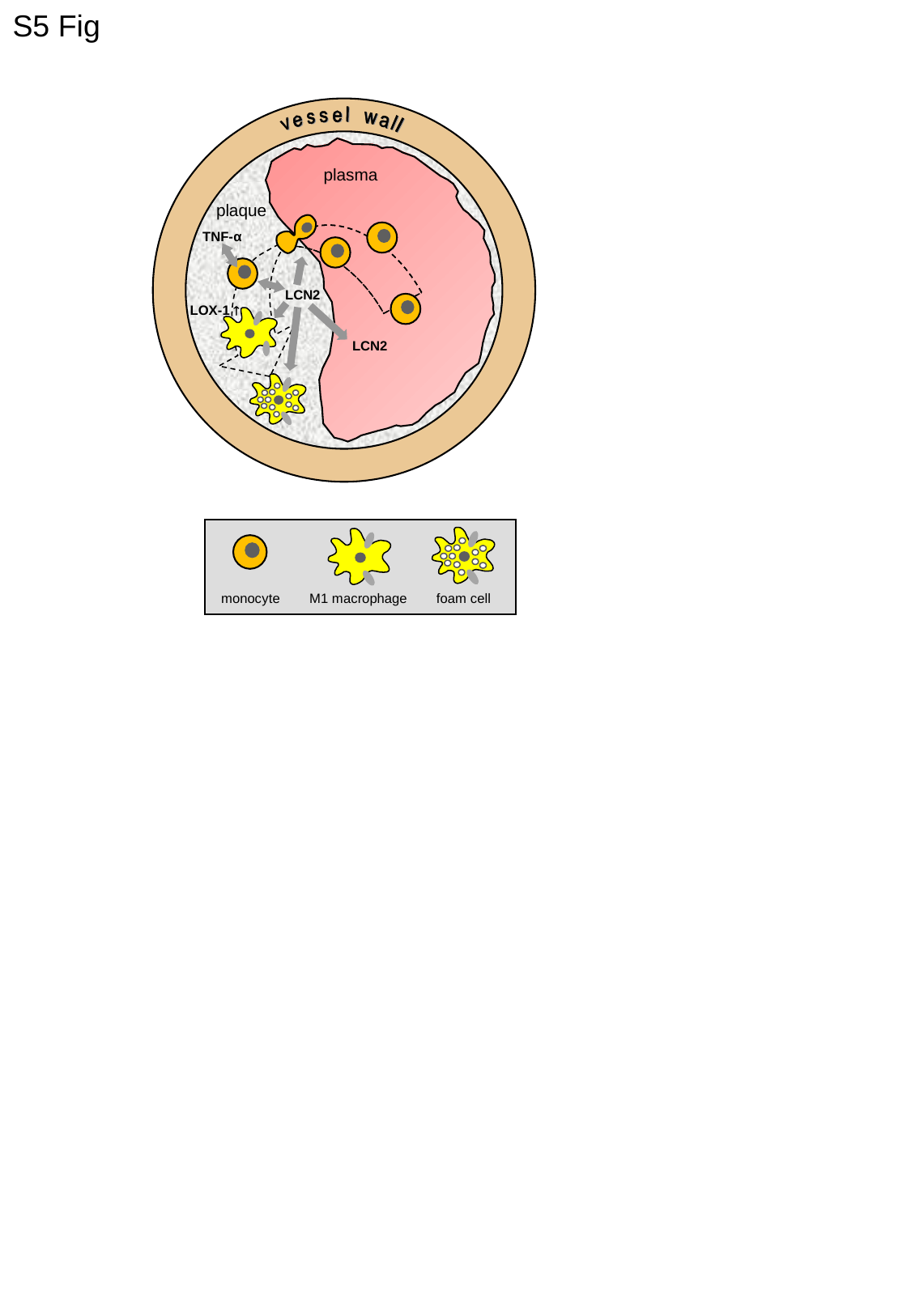

S5 Fig
plasma
plaque
LOX-1 
LCN2
TNF-α
LCN2
monocyte
M1 macrophage
foam cell

Supplement: S5 Fig — (PPT) [file pone.0137924.s005.ppt]
