# Supplementary material for: Lipocalin (LCN) 2 Mediates Pro-Atherosclerotic Processes and Is Elevated in Patients with Coronary Artery Disease
Source: PLoS One. 2015 Sep 14;10(9):e0137924. doi: 10.1371/journal.pone.0137924 (PMC4569430; doi:10.1371/journal.pone.0137924)
Supplement: S1 Table — Body-mass index is defined as the weight in kilograms divided by the square of the height in meters. Median; 25th/75th percentile, *P<0.05 vs. control, **P<0.01 vs. control, §§P<0.01 vs. CAD1, #P<0.05, ##P<0.01 vs. CAD2. (DOC) [file pone.0137924.s006.doc]

**S1 Table**: Characteristics of the patients.

Characteristic *group class-divided B*

*Control CAD CAD 1 CAD 2*  CAD 3

n 20 78 24 27 27

male/female (%) 45/55 73/27 71/29 67/33 81/19

age 61.5 (56.8-70.5) 69.0 (61.0-76.8) 62.5 (50.8-72.3) 69.0 (63.0-73.0) 73.0 (67.0-78.5)*^,§§^

Smoking status

Current smoker (%) 20 21 29 19 15

Former smoker (%) 40 54 25 62 70

Never smoked (%) 40 25 46 19 15

Body-Mass-Index 28.2 (26.4-32.0) 26.8 (24.3-29.4)** 26.2 (24.1-30.6)* 25.7 (23.8-27.9)** 27.8 (26.1-29.6)^#^

Blood Pressure

Systolic (mm Hg) 145.0 (135.8-157.3) 141.0 (130.8-161.3) 145.0 (130.8-174.0) 140.0 (131.3-160.3) 141.0 (130.3-151.0)

Diastolic (mm Hg) 83.0 (76.8-89.3) 82.5 (75.0-89.3) 84.5 (79.5-95.0) 81.5 (70.5-89.8) 80.5 (75.3-87.8)

Cholesterol (mg/dL) 182.0 (175.0-214.3) 193.0 (170.0-220.0) 197.0 (184.0-228.0) 213.0 (174.0-220.0) 159.0 (143.0-189.0)

hsCRP (mg/dL) 1.94 (0.77-3.16) 2.47 (1.02-4.08) 2.23 (0.72-4.20) 2.28 (0.88-3.71) 2.90 (1.32-6.86)

LCN2 (ng/mL) 51.2 (45.2-67.4) 70.4 (51.2-96.3)** 69.1 (45.2-93.7)* 60.5 (50.2-76.2)* 83.6 (60.0-111.2)**^,##^
