# Supplementary material for: Lipocalin (LCN) 2 Mediates Pro-Atherosclerotic Processes and Is Elevated in Patients with Coronary Artery Disease
Source: PLoS One. 2015 Sep 14;10(9):e0137924. doi: 10.1371/journal.pone.0137924 (PMC4569430; doi:10.1371/journal.pone.0137924)
Supplement: S2 Table — (DOC) [file pone.0137924.s007.doc]

**S2 Table**: Primer sequences for real-time PCR.

**Primer Sequence 5´− 3´ Amplicon size**

Arg1 forward CAG AAG AAT GGA AGA GTC AG 250 bp

Arg1 reverse CAG ATA TGC AGG GAG TCA CC

CCL5 forward CCA CTT CTT CTC TGG GTT GG 109 bp

CCL5 reverse GTG CCC ACG TCA AGG AGT AT

CD36 forward TCG GAA CTG TGG GCT CAT TG 314 bp

CD36 reverse CCT CGG GGT CCT GAG TTA TAT

HPRT1 forward CCT GCT GGA TTA CAT TAA AGC ACT G 352 bp

HPRT1 reverse GTC AAG GGC ATA TCC AAC AAC AAA C

IL-6 forward CCA TCC AGT TGC CTT CTT G 222 bp

IL-6 reverse AAG TGC ATC ATC GTT GTT CAT AC

iNOS forward CCA AGC CCT CAC CTA CTT CC 127 bp

iNOS reverse CTC TGA GGG CTG ACA CAA GG

LCN2 forward CCA GTT CGC CAT GGT ATT TT 102 bp

LCN2 reverse TCC TTC AGT TCA GGG GAC AG

LOX-1 forward ACC TGC ACT CCT TCT TCC CCT TTG T 159 bp

LOX-1 reverse GCC TTT GAG CCC TCT GCC TGC

SRA-1 forward CCG ACC TTA TAG ACA CGG GAC GC 114 bp

SRA-1 reverse TCC CAT GTT CCT GGA CTG ACG A

SRB-1 forward CTG CGC AGC CAG GAG AAA TGC 108 bp

SRB-1 reverse GGC AGC TGG TGA CAT CAG GA

TNF-α forward GTA GCC CAC GTC GTA GCA AAC 118 bp

TNF-α reverse CTG GCA CCA CTA GTT GGT TGT C

Ym1/2 forward TGG AAG TTT GGA CCT GCC CCG 125 bp

Ym1/2 reverse TGC CAG TCC AGG TTG AGG CCA
